# The Hospital Nursing Supplement

**Published:** 1894-12-15

**Authors:** 


### "The Hospital" Nursing Mirror.

Being the Extra Nursing Supplement of "The Hospital" Newspaper.

[Contributions for this Supplement should be addressed to the Editor, The Hospital, 428, Strand, London, W.C., and should have the word "Nursing" plainly written in left-hand top corner of the envelope.]

### News from the Nursing World.

### OUR CHRISTMAS COMPETITIONS.

We have made arrangements for the reception of a great many parcels this year and look forward to the pleasant labour of distributing next week a quantity of useful clothing to sick folks in hospital wards. Will our readers please remember that by Tuesday, 18th inst., all articles intended for competition should reach the office addressed to "Nursing," care of Editor of The Hospital, 428, Strand. In all cases the names and addresses of the senders should be enclosed.

### A NEW NURSING HOME.

H.R.H. the Duchess of Albany opened the new Home of the Kingston-on-Thames Nursing Association on the 3rd inst. The excellent work accomplished since its first commencement, twelve years ago, has been duly appreciated in the district, and the growth of the new buildings has been watched with great interest. Purses were presented to the Duchess at the conclusion of the ceremony, after which she made some purchases at the bazaar held in aid of the funds of the Association.

### LACKING DISCRETION.

In a letter on another page, attention is this week called to the reprehensible practice of a nurse discussing operations and other strictly professional matters in public. Our correspondent is not singular in her experience, for similar confidences may frequently be heard in crowded vehicles. The vulgarity and bad taste evinced by these remarks augur ill for the unfortunate patients left to the care of the speakers. Our correspondent's other complaint is equally well founded, for ill-timed levity is the natural accompaniment to the startling appearance of the worse type of modern nurse. Flirtations in uniform, frizettes, paint, powder, high-heeled shoes, and jangling chatelaines are all abominations in the eyes of the true and earnest nurses who, we are fain to believe, still constitute the greater as well as the more influential section of their profession.

### THE TRAINED NURSES' CLUB.

In spite of the heavy rain on Friday many visitors on that and the preceding day found their way to the sale of work at the Trained Nurses' Club in Buckingham Street. The articles exhibited were chiefly of a useful description, but this did not prevent their being also exceedingly pretty. Those who organised the sale and the excellent entertainments which took place at intervals may well congratulate themselves on the social and financial success of their undertaking.

### A TEST CASE.

"It's much easier to write indignantly to the papers than to rouse the committee," retorts the probationer with a grievance, scornfully incredulous of the assertion that the second is the more honourable procedure. At a large general hospital the wiser step has however been recently taken by a large proportion of the ward sisters and nurses (not that proverbial grumbler, "the new pro"!), who have signed their names to a petition for the revision of certain details. Such a statement cannot be lightly set aside by those responsible for the management of each department of the institution, and the courageous action of the workers in applying first to those from whom they have a right to demand justice, must necessarily secure a fair inquiry into the points at issue. If properly and promptly conducted, this step may obviate the need for a journalistic controversy, which appears otherwise imminent.

### PAUPERS AND PEERS.

"The life of a pauper must be attended to as much as the life of a peer," was the courageous utterance of the chairman of the Kidderminster Board of Guardians, reported last week in the local press. The nursing question, as dealt with in that district, will form valuable history for the next century, although students may wonder why a cheer should have rewarded Mr. Longe, the Local Government Board inspector, for, shaking his head, as he is said to have done, at the chairman's statement that "the medical officer in his professional capacity dominated over the Board." But the cheers, considered by serious-minded folks a little misplaced, were sufficiently characteristic of gentlemen who consider that typhoid fever does not require trained nursing. A confession that "with the drainage of the house in its present condition it was liable to spread the disease all over the establishment" seems to us strong evidence of the efficiency of the measures by which the medical officer has been instrumental in preventing an epidemic.

### GOOD WORKS WELL DONE.

An excellent example in practical usefulness has been set by the lady guardians at Toxteth Workhouse in two important departments. In making known the existence of bookshelves in the nurses' sitting-room, they also call public attention to the fact of their being at present empty, and urgently appeal for gifts of histories, biographies, wholesome stories, and novels; books on nursing and technical subjects will doubtless be equally acceptable. For twelve months also patience and time have been freely devoted by two of the women guardians to cases in the maternity wards, with the result that out of fifty-nine girls reported on, forty are now doing well, through the kindly personal influence which has been so unstintedly exercised by these competent workers.

### QUEEN'S NURSES AT WOLVERHAMPTON.

The Home for the Wolverhampton Branch of the Queen's Jubilee Institute, which was opened on November 28th, materially adds to the comfort of the nurses. The house, rented by the Committee, is in St.

Paul's Terrace, and has been nicely fitted up and furnished. The formal opening ceremony was performed by the Mayoress, Mrs. Mender, who wished the Home every success. A thorough inspection of the rooms was made by the guests, and appreciation of the work already accomplished by the district nurses was cordially expressed.

### FRIENDLY CO-OPERATION.

The miners at New Seaham and at Silksworth collieries are most active supporters of their respective district nurses. The presence on the committees of representatives of those for whose service the associations exist has resulted in universal support being given to Lady Londonderry's excellent scheme. She presided at meetings recently held in both districts, and with regard to the Convalescent Home now under consideration, her ladyship said to her earnest co-workers, "it is a matter for your own consideration." One of the miners made an excellent speech in which he acknowledged the value of the work of trained district nurses.

### GOOD NURSES APPRECIATED.

A pleasant afternoon meeting was held at the Taunton and Somerset Hospital on the occasion of the distribution of prizes to the nurses. Courses of lectures have been given by Dr. Macdonald, Dr. Bain, and the matron, Miss Harris, and the subsequent examinations showed most satisfactory results. Dr. Liddon saw cause for congratulation in the completion of the wing, which made it practicable for the nurses to sleep outside the hospital—an arrangement conducive to their health and well-being. He also spoke of the ability of Miss Harris—"a lady who seemed thoroughly imbued with the spirit of work which Miss Wilson had shown." It is therefore evident that well-trained nurses are appreciated at Taunton, for the Mayor added his testimony, saying, "Far from being a trouble, these nurses saved trouble in every possible way."

### A CALAMITY AVERTED.

Thanks to the presence of mind of the members of the nursing staff at Montrose Royal Infirmary no panic followed the outbreak of fire in one of the newly-painted surgical wards. Smoke having been observed, the prompt removal of a mantelpiece by Firemaster W. Craigie revealed a burning joist. Although the danger was rapidly averted, considerable injury was done to the ward. The nurses ably seconded the measures taken to subdue the fire, and assisted in the transfer of the patients to other parts of the building.

### TORONTO GRADUATES.

The Lady Superintendent of the General Hospital Training School, Toronto, presented her annual report on the 28th ult. to a large assemblage of interested visitors. Two hundred and two nurses have already completed the course, and there are at present fifty pupil and four graduate nurses in the Training School. Blue dresses, white aprons, and caps constitute the uniform worn by the thirty young ladies admitted each year. Addresses were afterwards given by Dr. Charles Sheard, Dr. Cassidy, and Mr. James Beaty, Q.C.

### A GOOD CAUSE.

The Doll Exhibition, in aid of the Society for the Prevention of Cruelty to Children, at 20, Molesworth Street, Dublin, was opened on Tuesday, 4th inst., by Lady Victoria Hamilton, the prizes being distributed by the little daughter of His Excellency the Lord Lieutenant. Those for the best dolls dressed as hospital nurses in the 2nd and 3rd divisions respectively have been awarded to Miss Gladys Grimshaw and to Miss Darwell. Prizes have been presented by T.R.H. the Princess Christian of Teck, the Duchess of Albany, and others. The society is badly in need of funds, and it is hoped that the exhibition has proved a brilliant financial success.

### JOURNAL CLUBS.

The Journal Club in connection with the Johns Hopkins' Training School, to which reference was last week made in our columns, is a movement which we should like to see extensively adopted. Long ago the idea was suggested by us as both feasible and desirable, and we should be glad at any time to give all possible assistance to institutions willing to follow the excellent example now set by our American Sisters. Discussions on articles which have appeared in current nursing literature in all countries are encouraged. The subjects are chosen and speakers selected beforehand, the scheme formulated in America covering six or seven months. This gives nurses time to study thoroughly the various subjects, besides promoting systematic reading by the members of the club. To keep well up to date in nursing progress at home and abroad is surely a desirable ambition in England as well as America.

### SHORT ITEMS.

Some Irish members of the General Medical Council were present on December 1st at a special meeting of the Executive Committee of the R.B.N.A., Princess Christian being in the chair. Although the medical gentlemen expressed interest in the objects of the association, they appear to have been unanimous as to the existence of the difficulties rendering it undesirable that any immediate effort should be made to establish a branch in Ireland.—The Bath Guardians have left the consideration of nursing matters at their infirmary as a legacy to the new Board.—The Rev. A. D. Freeman spoke at a recent meeting of the Steyning Guardians in commendation of the work and influence of Miss Mowatt, head nurse in their infirmary. It was agreed to raise her salary from £40 to £60 per annum.—A grand concert, held at Milton Court, resulted in a sum of £70 being handed to the Dorking Nursing Association.—The third annual meeting of the Milngavie Nursing Association was held the other day in the Burgh Hall.—Miss Marwick succeeded Miss Foulkes as Queen's Nurse at Forfar, and took up her duties on November 16th.—The first banquet of the Chicago Medical Woman's Club took place last month, Dr. Gertrude G. Wellington being Chairman of the Arrangement Committee.—Owing to pressure of other duties, Miss C. J. Wood has been obliged to resign her position on the Executive Committee of the Workhouse Infirmary Nursing Association.—The ordinary general meeting of the Glasgow Sick Poor and Private Nursing Association took place recently, and the nineteenth annual report was presented.—A fire broke out at St. Pancras Infirmary, Highgate, on Monday morning last, but was happily subdued without alarming the patients.

### The Dietetic Management of Infancy.

By George Dickson, M.B., C.M.

II.—CHANGE OF FOOD.

The infant should be weaned when it is about twelve months old. Boiled cow's milk should gradually take the place of the breast meals, although the latter should be given for one week twice daily, and for a second once daily, before being stopped altogether. Up to eighteen months cow's milk should continue to be looked upon as the chief article of diet, and anything else merely as an addition to it.

From eighteen months to two years a little pounded mutton, chicken, or beef may be given, and for a variety a choice of the following articles: Mashed potatoes or cauliflower with gravy, milk or custard puddings, bread and butter, and fruit. Long after two years the simplest food is the best. Porridge and milk constitutes an almost ideal food for infants, and should largely enter into the dietary of every child.

Coffee and tea are best avoided, but cocoa, especially if made with milk, is a nutritious and harmless beverage. When the teeth have fully developed, food requiring mastication should be given.

It should never be forgotten that children are liable to thirst as well as adults, and this is best relieved by cold water, toast and water, or thin barley water.

Artificial Feeding.

If, for any reason, the mother be unable to suckle her child, the best substitute is a selected wet nurse. This is, however, expensive, and often impracticable. And the same difficulty arises with regard to asses' and goats' milk. So that in ninety-nine cases out of every hundred the infant has to be brought up on cow's milk.

Cow's milk differs from human milk in several important points, which are roughly indicated in the subjoined table:—

| | Cow's milk. | Human milk. |
|---|---|---|
| Reaction ... ... | Acid | Alkaline |
| Proteid (Casein) ... | 4·2 | 2·7 |
| Fat (Butter) ... ... | 3·8 | 3·5 |
| Sugar ... ... ... | 3·8 | 5·0 |

There is a slight difference in salts, which is unimportant.

It will be noted that cow's milk contains more casein or curd and less sugar than human milk. It is not the larger quantity of the casein, however, that causes cow's milk frequently to disagree with babies, but its indigestibility, as compared with the casein of human milk. When taken into the stomach the casein is in both cases coagulated by the action of the gastric juice, but the curd of human milk forms itself into fine flocculent particles, while the curd of the cow's milk runs together into large cheesy indigestible masses. It is these cheesy masses that are so disastrous to the tender stomachs of infants, and so frequent a cause in them of gastro-intestinal disorder.

It ought to be clearly understood that any amount of dilution of cow's milk, provided only plain water is used, fails to produce any alteration in the digestibility of the casein, which still coagulates as before.

To make the milk of the cow more closely approximate in composition and character to human milk it is obvious that it must be diluted and sweetened, and something added to cause the casein to separate into more digestible particles. The proper dilution for infants under one month is one in four, and above this age one in three. To effect a change in the coagulation of the casein there are various expedients, four of which are in general use. (1) Boiling the milk. (2) The addition of barley water to the milk. (3) The addition of lime water to the milk. (4) The addition of gelatin and water to the milk. Boiling causes the curd to separate into finer masses, but does not of itself produce a sufficient change in this direction, so that it requires to be supplemented by one of the other methods. Milk for babies should always first be boiled, as by this it is sterilised, and the danger of its conveying disease is obviated. Barley water should be freshly made, as it rapidly grows sour. It acts mechanically by separating the particles of casein from each other. Lime water, to be effectual, must always form at least one-third of the mixture employed. It acts by neutralising the acidity of the gastric juice, and thus preventing clotting of the curd in the infant's stomach altogether. Gelatin acts, like the barley water, mechanically, and is prepared as follows: A teaspoonful of pure gelatin soaked in half a tumblerful of water for three hours should be turned into a teacup and stood in a saucepan of water boiling till dissolved. When cool it gelatinises, and about a teaspoonful should be added to each half-bottle of milk suitably diluted with plain water. Barley water has the advantage of adding in nutritive value to the food.

### Our Christmas Number.

Next week we publish the Christmas Number of The Hospital, and, with the kind co-operation of our friends, intend that the "Mirror" shall be especially attractive this year. H.R.H. the Princess of Wales, the much-beloved President of the Royal National Pension Fund for Nurses, will be the subject of our illustration; and we believe that our nurse readers and others will welcome the possession of so excellent and charming a portrait. As usual, we mean to give particulars of Christmas festivities projected within hospital walls. Bright and pleasant books for Christmas reading will be introduced to those who have little time t› seek these for themselves. A special article on "Where to go for Christmas and New Year's Gifts" will save much time to purchasers, and help them to secure what they want at once. With view of making the Christmas Supplement as useful and popular as possible, it will be considerably enlarged.

### A Present for Christmas.

Any reader in doubt as to what to give as a present to heads of families, the clergy, or to anyone interested in health questions or the sick, should purchase a copy of "Helps in Sickness and to Health," which the Scientific Press, 428, Strand, W.C., have just issued. The *Times* says, "It would be difficult to find a book which should be more welcome in a household than this most useful book," to which we may add that every nurse, and especially every district and private nurse, should keep "Helps in Sickness and to Health" as her constant companion. The published price is only five shillings, and we hope it may prove, as it deserves, the most popular of Christmas and New Year's gifts.

### Appointments.

Anglesey and Carnarvonshire Infirmary, Bangor.—Miss Poynter, who was trained at the Royal Infirmary, Liverpool, has been appointed Lady Superintendent of the Anglesey and Carnarvonshire Infirmary. Miss Poynter adds a knowledge of the Welsh language to her other qualifications for this post, and takes many good wishes with her.

St. Bartholomew's Hospital, Chatham.—Miss Flora Prosser has been appointed Matron of this hospital. She was trained at the Northern Hospital, Liverpool, and afterwards held the post of sister at St. Marylebone Infirmary. Miss Prosser was afterwards matron of the Zenana College, London, and matron at Tewkesbury Hospital. We congratulate her on her appointment.

### The Royal British Nurses' Association.

### BAZAAR AND CONVERSAZIONE.

A bazaar in aid of the funds of this association was opened by Her Royal Highness Princess Christian, the president, on December 6th, 7th, and 8th, in the Grafton Galleries. On Thursday, the opening day, Princess Christian, accompanied by Princess Victoria of Schleswig-Holstein, and attended by Miss Emily Lock and Baroness Zund Von Egloffstein, was received at the entrance to the Galleries by the principal members of the committee. Her Royal Highness was presented with a magnificent shower bouquet, a similar one being given to Princess Victoria. The band of the Royal Horse Artillery was stationed in the vestibule, and greeted the entrance of the royal party with a flourish of trumpets, the Blue Zouave band striking up the National Anthem, whilst Her Royal Highness passed to the daïs, on which her stall was situated. After a few introductory remarks by Sir James Crichton Brown, Princess Christian declared the bazaar open, and the business of the day commenced. The hall presented an unusually gay appearance, all the stalls being draped in red, blue, and white, and decorated with plants. The President's stall overflowed with beautiful and interesting articles. Chief among the latter were recent photographs of Her Majesty the Queen, which found a ready sale. The monograph written by H.R.H. Princess Christian on the work of the association for the International Congress at Buda Pest, had been reprinted on Old English paper, each copy bearing her autograph. On either side of the daïs were the flower and the dairy stalls, the former presided over by Mrs. Arthur Bouchier, and the latter under the direction of Miss de Pledge. One of the chief attractions in connection with this stall was the process of separating cream and making it up into butter by Miss Williamson and Miss Gibbons, the former being one of the County Council's demonstrators. The Aylesbury Dairy Company contributed excellent milk and cream cheeses, which were in great request, also fresh eggs and country butter. The general and special hospitals' stall occupied the left-hand side of the room, and were under the charge of Miss Mollett, Miss East, and others. Opposite stood the Children's, St. George's, and the Infirmaries' stalls, under the superintendence respectively of Miss Anderson, Mrs. Coster, Miss F. Hughes and her assistants. The "private nursing" stall was presided over by Mrs. Bedford Fenwick, and the Middlesex Hospital stall by Miss Thorold. Entertainments and exhibitions filled up the programme, and were largely attended, Princess Christian being indefatigable in her efforts each day.

### The Conversazione.

On Friday evening the association held its annual conversazione at the scene of the bazaar. At nine p.m. Princess Christian was received at the entrance by the committee and honorary officers. She wore the gold badge of the Association and several orders, passing up the hall, through a double line of nurses, with whom the handsome accoutrements of the trumpeters of the Royal Artillery band, formed a brilliant contrast. Her Royal Highness presented the badge of the association to each newly enrolled member, and afterwards walked round the galleries and honoured with her presence a concert which was given under the direction of Mdlle. Marie Wolaska by some well-known vocalists, accompanied by the Blue Zouave band. The attendance this year was larger than usual, every variety of costume was to be seen, from the navy blue of the sister to the grey or blue of the probationer. A slight improvement was noticeable generally in the appearance of the nurses over last year, but many were still very far from being neat or professional-looking. The tendency towards wide, fashionably cut sleeves, touzzled fringes, and enormous "buns," was also marked, and, in regarding them one was tempted to quote Burns:

> Oh, wad some power the giftie gie us
> To see ourselves as ithers see us.

The bazaar was kept open during the evening, and an ample repast was served in the banqueting hall. Among the company present were Sir Dyce and Lady Duckworth, Sir Francis and Lady Jeune, Sir Richard Webster, Dr. and Mrs. Coupland, Dr. and Mrs. Buzzard, Dr. and Mrs. Heron, the Hon. Mrs. Randolph Clay, and Miss Lowe.

The nurses as a whole had a far less trim and professional air, as we have noticed on former occasions, than their sisters presented at the annual gathering of the Workhouse Infirmary Nursing Association. The appearance of the Chelsea Infirmary nurses provoked many eulogistic remarks from visitors to the Grafton Gallery, who were agreeably impressed by their neatness.

[The above report is supplied by our Special Correspondent, as we were struck by the inaccuracies which appeared in the accounts sent to the daily papers. In the *Times* it was stated, for example, that Sir Sydney and Lady Waterlow were present, though both of them are, we understand, in the South of France. It would seem as if the writer of the *communiqne* in question had simply written down the names of every personage connected in any way with the association, and issued the list to the Press as that of those present. It is due to the Press that some explanation from the Executive Committee should be promptly forthcoming.]

### Notes from India.

### THE RANGOON STATION HOSPITAL.

In 1891 the Indian nursing service was extended to Burmah, and the headquarters made at Rangoon. Later on an offshoot was opened at Mandalay. Rangoon is situated on the coast, and is a curious old place, full of many odd buildings and temples, and in the native bazaars much that is curious and valuable may be picked up. New Burmese silver workmanship is lovely, and easily recognised by the perfection and boldness of the designs; but even these fade into insignificance beside the old Burmese treasures, which may be collected in out-of-the-way corners. Burmese "Bibles" fetch prices from Rs. 15 to Rs. 300. Some are made on strips of palm leaves, with the characters stamped in, and threaded together with cane straws, and bound in pieces of bamboo. I believe these "Bibles" teach the Buddhist faith.

The Station Hospital is a fine two-storied building, and the block in which the sisters work contains forty-six beds, thirty downstairs and sixteen above. The latter floor is divided into small wards, with lattice work verandahs. There are handsome staircases; and an excellent officers' ward next the sister's duty-room is well furnished and contains two beds. The sisters have a large comfortable bungalow some two miles from the hospital, and they go backwards and forwards in a gharry, sometimes drawn by bullocks, taking about thirty minutes over the distance It is a very humid climate. "The rains" last about six months, making it damp and muggy, the thermometer registering from 79 deg. to 85 deg. Fahr. during that time. The early mornings, however, are cool, and on the whole Rangoon is not unhealthy for the troops, but the enervating climate tends to unfit them for service in other parts of India. Last summer only one sister was working at the hospital, namely the superintendent. When the staff is complete, however, it consists of a superintendent and three nursing sisters; but Mandalay being supplied from there, and the sisters requiring occasional leave, work became very hard for a lady left to work alone. Her duty lasted from 6.30 a.m. to 10.30 p.m., with only an hour's interval for a drive in the evening. There are plenty of shops, native and European. Living is very dear, servants' wages being double what they are in India, and the price of mutton four times as much. The sisters must find it hard to make both ends meet. There is a good civil hospital at Rangoon, where Europeans are treated.

### Where to Go.

London Hospital.—Children's Christmas Trees, Monday, December 31st; Royal Punch and Judy Show at half-past two. Visitors are cordially invited to inspect the wards and Nursing Home.

### The American Woman at Home.

By Our Own Correspondent.

### IV.—THE DRESS OF SOME AMERICAN WOMEN.

I would certainly like to have a license of several thousand words more than the editor allows me when I attempt to deal with a subject of such immense importance as the dress of the American woman. It is the object of her deepest meditations, and her most ardent desires. She spends on it more than double the money an Englishwoman, even an extravagant Englishwoman in the same position, would do, and shows a perfect genius for hurling together contrasting colours and opposed materials in a fashion that would scare the most *chic* Parisienne. She professes to be guided by the modes of London and Paris, but she outruns her teachers, and is always a little more than ahead of the fashion. The reserve, the avoidance of extremes, to which we cling, seems to her mere dowdiness. English clothes, she says, are "homely." And, alas! she has degraded this beautiful word, so full to us of kind and tender meaning, of cherished memories, or cherished hopes, to mean only ugly, dull, unattractive. The American wants to be striking, obvious, notable; that the woman should be more than her dress seems to her a poor ideal. She wants to be different from her neighbours, and you can get infinitely more difference in gowns than in mere eyes and noses. There is a tradition, fondly believed by others than herself, that the American woman is always well dressed. Certainly she is always smartly clad, but she too often lacks that perfect suitability of the gown to the occasion of wearing it which forms the European ideal, and the conscious air of "wearing"—"You Americans 'wear' even your eyes," Mr. Henry James makes one of his characters say—vitiates the general effect. Plaids that Scotland never knew, fur garments trimmed with lace and bright-hued velvets, daring combinations of blue with cherry colour—these things are conspicuous, but are they beautiful? One merit, from the American point of view, they have; they are so conspicuous that they can be worn but a few times; therefore they are expensive. This counts for something in a country where, as it seems to us invention is ransacked to find new ways of spending wealth too rapidly acquired, where silver and gold seem to be of as little account as they were in Jerusalem in the days of King Solomon. In the accessories of dress you may do a good deal of spending; even a mere man may succeed in this if he adds to his luxuries a silver-mounted moustache comb at a couple of guineas, or a corn knife that will cost ten pounds. And if this is possible to a man what may not his wife attain to, beginning at those simple necessaries of life, a pair of diamond earrings and going on through every article of woman's wear?

Tell it not in our modest Philistia, but you will see in Tiffany's garter buckles of diamonds, and I feel sure that at least the mother of the Duke in the Bab Ballads, who

"Wore a pair of golden boots
And silver under-clothing,"

must have hailed from the other side of the Atlantic. For the American woman's luxury is no superficial thing, it goes through every stratum of her adornment, and you will find people in modest positions and otherwise quite unpretentious in their mode of life, buying under garments of the daintiest silk or finest batiste, elaborately trimmed with lace and ribbons, such as we should think suitable only for the trousseau of a peer's daughter. Another characteristic of American dress is that old women and young dress equally gaily. If the middle-aged American dame cannot flatter herself that, like Cleopatra, "age cannot wither her," she at least ignores its ravages, and takes good care that custom shall not stale the infinite variety of her costumes. Twenty and fifty wear the same style of gown—smart checks, vivid reds and blues, if they are in fashion, transparent muslins and chiffons. The climate in summer certainly commands thin clothing, but surely there are black gauzes and grenadines that form a better setting to a faded face than white or coloured muslins, or the gayest and most fanciful patterns of foulard and china silk. But, perhaps because the young girl forms so important a part of American society, the American woman is determined to be a young girl to the end of her days. She is youthful enough in her ways, it must be admitted, as fond of gaiety—not mere pleasure-seeking but absolute romping—as her daughters, or more so, if these damsels have a touch of the fashionable Anglo-mania. I have heard of two ladies, one a grandmother, the other the mother of a grown-up daughter, who amused themselves by playing Aunt Sally with a dinner service belonging to one of them which she had tired of. You can't expect giddy girls like that, even if they do happen to be nearer sixty than fifty, to dress like our English dowagers. One lady, about to visit a daughter who had married and settled in England, asked me quite pitifully if she would be obliged to comply with British custom to the extent of wearing a cap. I assured her that the adornment in question was by no means so obligatory as she seemed to think, though for my own part I believe some soft arrangement of lace and pale-hued ribbons or feathers would have added to the charm of her pretty white hair.

The conventions of American life are so different from ours, and in their own way quite as binding, that even their most elaborate costumes are apt to strike us as unsuitable to the occasion. That a lady should go to an afternoon party with a gown even slightly décolletée strikes us as remarkable, yet in spite of the occasional strictures of fashion journals, which quote the laws of Paris as supreme, the fashion is regarded as quite permissible; and the same dress will be looked upon as equally suitable for evening wear. In fact there is no rule of high or low, of morning or evening dress. Let the garment be but gay enough, and there is hardly an occasion for which it will not do. The result is that to an English eye an American appears over-dressed at lunch, and under-dressed at dinner. The method has certainly the advantage that the gown which will no longer bear the light of day can be transferred to evening wear without change of style. Not that this will be its fate. There will be some re-arrangement of sleeves and flounces, some of that endless "fixing over," which occupies so much of an American woman's time, and has created a race of really skilled seamstresses which we might profitably try to borrow. And ladies, both old and young are themselves both more willing and more able to help in the work than we are. Whether one cannot put one's time to better account than in endlessly altering one's gowns is another question; one cannot deny the skill and perseverance with which it is done.

As with dresses, so with jewels. There is no occasion when diamonds may not be worn, rings by the score, and brooches by the dozen. And considering the artistic designs of most American jewellery one can hardly condemn the fashion. Our goods for the most part look heavy and clumsy beside their graceful creations, and this very grace takes away the appearance of ostentation which the display would otherwise bear.

### Presentations.

On her departure from the Royal Hospital for Sick Children, Edinburgh, with which she had been connected for seven years, Miss Bird, the matron, was presented with a silver tea service by the nurses, a handsome travelling clock from the Ladies' Committee, and a brass-mounted oak writing desk from the servants.

### Wants and Workers.

Nurse B. would be glad to hear of a Home or other Institution where a poor girl (aged 16) with a curved spine could be received for calisthenic treatment under medical supervision.

### Everybody's Opinion.

[Correspondence on all subjects is invited, but we cannot in any way be responsible for the opinions expressed by our correspondents. No communications can be entertained if the name and address of the correspondent is not given, or unless one side of the paper only be written on.]

### MIDWIVES OR ——?

"A Trained Nurse" writes: I have read with dismay the report of the resolution of the General Council of Medical Education, and as usual I turn to The Hospital for help and advice. In telling other nurses that they could not fairly consider their training complete without going through a course of midwifery and securing the L.O.S., I have hitherto only acted like other experienced nurses. Now I want to know, please Mr. Editor, exactly what effect this action of the Council will have on us? It seems to me that the uncertificated "anomaly," who poses as a "midwife and monthly nurse," is going to have a good time. She already goes her way unmolested, except when now and then a coroner's remarks shed a temporary gloom over her career. As far as I can judge, being only an illogical Woman (not entitled to the prefix of New !), that the trained nurse, whose full course of instruction in midwifery has hitherto been guaranteed by the "diploma," must in future be content with the same status as the woman whom circumstances alone have taught a little dangerous knowledge.

[The following remarks, by one thoroughly conversant with the subject, have just reached us, and we imagine that they constitute a sufficient reply to our correspondent's letter.—Ed. *T. H.*]

"The General Medical Council has declared that many of the certificates granted to midwives are "colourable imitations" of medical diplomas. We are very glad it has done so, as this will clear the air of some of the thunder-clouds which have tended to obscure the true value of a properly trained and certified midwife, and the prospects of a Bill for the registration of midwives in the immediate future have improved to a corresponding extent. We regret much that it should have been felt necessary to single out the certificate issued by the London Obstetrical Society as one to be made an example of. In itself the certificate is far more modest and unpretending than that issued by many of the other examining bodies, and the examination was instituted many years ago on philanthropic principles, and till within the last few years was carried on gratuitously. It is recognised in all parts of the country as indicating the holder's knowledge and training, and we trust that the society will see its way to modify it in accordance with the demands of the General Medical Council."

### AT THE BAZAAR.

"Trained, but Unregistered" writes: May I call your attention to the obvious injustice which has been done to many hospitals by the reports that the pretty bazaar at the Grafton Galleries was in any way representative of "most of the metropolitan hospitals." I found myself wandering round like a lost sheep amongst "specials" and "children's." Except "the Middlesex," I found the name of no metropolitan general hospital placed over a stall, and I came away sympathising with the gentleman who complained most audibly in the doorway of having been led to waste valuable time in searching for the non-existent stall of "St. Thomas'."

### ILL-TIMED CONFIDENCES.

"The Matron of a Large London Hospital" writes: I overheard with great surprise and regret a conversation between two nurses (in uniform) in an omnibus going from Charing Cross along the Edgware Road on the morning of the 2nd inst. The following is the gist of the conversation: The nurses discussed the sister under whom they worked; the appointments and flirtations they themselves had with certain medical men, whose names they mentioned. They gave details of various operations and dressings, which must have been trying and offensive to the other passengers. The name of a celebrated Birmingham surgeon was also mentioned, and his work criticized. If nurses behave in such an indiscreet manner, it is, indeed, no wonder that the public speak disparagingly of them. I sincerely hope this letter will fall under the notice of the two nurses, and that it may teach them to be more discreet in future, avoiding the discussion of hospital affairs in public places. It is to be hoped that nurses in general have more respect for themselves and their profession than to follow such an odious example. It is conduct like this which brings discredit on what should be a high and noble calling.

### MEDICAL OFFICER AND MATRON.

"An Old Nurse" writes: Having read in The Hospital the opinions of several writers respecting the medical officer's and matron's duties, I venture to send a few lines on the subject. I am a very old matron, and have been sister and head nurse in my time. The medical officer was always responsible for his patients being properly attended, but when a matron was also a nurse she was held responsible for the ward-nursing in every way. She has the power to make reports to the board of guardians if duties are neglected. A lady superintendent is truly the head.

### IRISH DISTRESSED LADIES' FUND.

The Marquis of Waterford writes from Curraghmore, Ireland:—Now that Christmas is approaching I hope you will again allow me, as chairman of the London committee of the above fund, to trespass upon your space in order that I may point out to the public how urgently subscriptions are required in support of this most deserving charity. The Irish Distressed Ladies' Fund was established for the purpose of giving assistance to ladies whose incomes were entirely derived from Irish land, and who have been reduced from comfort to penury by the recent agrarian troubles in Ireland, with which agrarian troubles, however, they have had absolutely nothing to do. The charity is still in need of funds in order to continue its good work, and to relieve the pressing and terrible distress that exists among a large number of ladies who are almost entirely dependent for existence upon what they receive from this association. During 1894 one hundred and eighteen ladies who, from age and infirmities were unable to work, received pensions according to the necessities of each case; twenty-five children are being educated; many invalids have received material assistance, and a large number of ladies have been employed in executing work, which has been sold at the English and Irish depôts of the charity, and at sales organised by the Scottish auxiliary committee. Every case is thoroughly investigated, and great care taken to give assistance only to those who are really deserving. I hope, sir, you will allow me also to say how grateful the committee feel to those who subscribed to this fund last Christmas and during the past year. The charity is entirely dependent on voluntary subscriptions, and, unless the public subscribe as liberally as heretofore, the aid given to these poor ladies will have to be materially reduced. The secretary will thankfully receive and acknowledge any subscriptions or donations, at the office of the charity, 17, North Audley Street, London, W., which is also the depôt for the sale of the Irish Distressed Ladies' work.

### PRIVATE NURSING HOMES.

Mrs. Stubbs, of Beresford House, Beaumont Street, writes: Having seen in last week's Hospital a comment on the expensiveness of existing nursing homes in contrast to the lower terms offered to private patients in one just opened . . . I think it is only fair to let your readers know that in this and, I believe, in many others, patients are received at from three guineas a-week in private rooms, with every modern convenience of electric light, &c.

### A CORRECTION.

Miss Barbar writes to say that she is at present Assistant-Matron at "Chortham" not "Chatham" County Asylum. The error was due to the word being somewhat indistinctly written.

